# Strategies to Enhance Periplasmic Recombinant Protein Production Yields in *Escherichia coli*

**DOI:** 10.3389/fbioe.2021.797334

**Published:** 2021-12-14

**Authors:** Alexandros Karyolaimos, Jan-Willem de Gier

**Affiliations:** Department of Biochemistry and Biophysics, Stockholm University, Stockholm, Sweden

**Keywords:** *Escherichia coli*, periplasm, recombinant protein, protein production, production optimization

## Abstract

Main reasons to produce recombinant proteins in the periplasm of *E. coli* rather than in its cytoplasm are to -i- enable disulfide bond formation, -ii- facilitate protein isolation, -iii- control the nature of the N-terminus of the mature protein, and -iv- minimize exposure to cytoplasmic proteases. However, hampered protein targeting, translocation and folding as well as protein instability can all negatively affect periplasmic protein production yields. Strategies to enhance periplasmic protein production yields have focused on harmonizing secretory recombinant protein production rates with the capacity of the secretory apparatus by transcriptional and translational tuning, signal peptide selection and engineering, increasing the targeting, translocation and periplasmic folding capacity of the production host, preventing proteolysis, and, finally, the natural and engineered adaptation of the production host to periplasmic protein production. Here, we discuss these strategies using notable examples as a thread.

## Introduction

**FIGURE 1:**
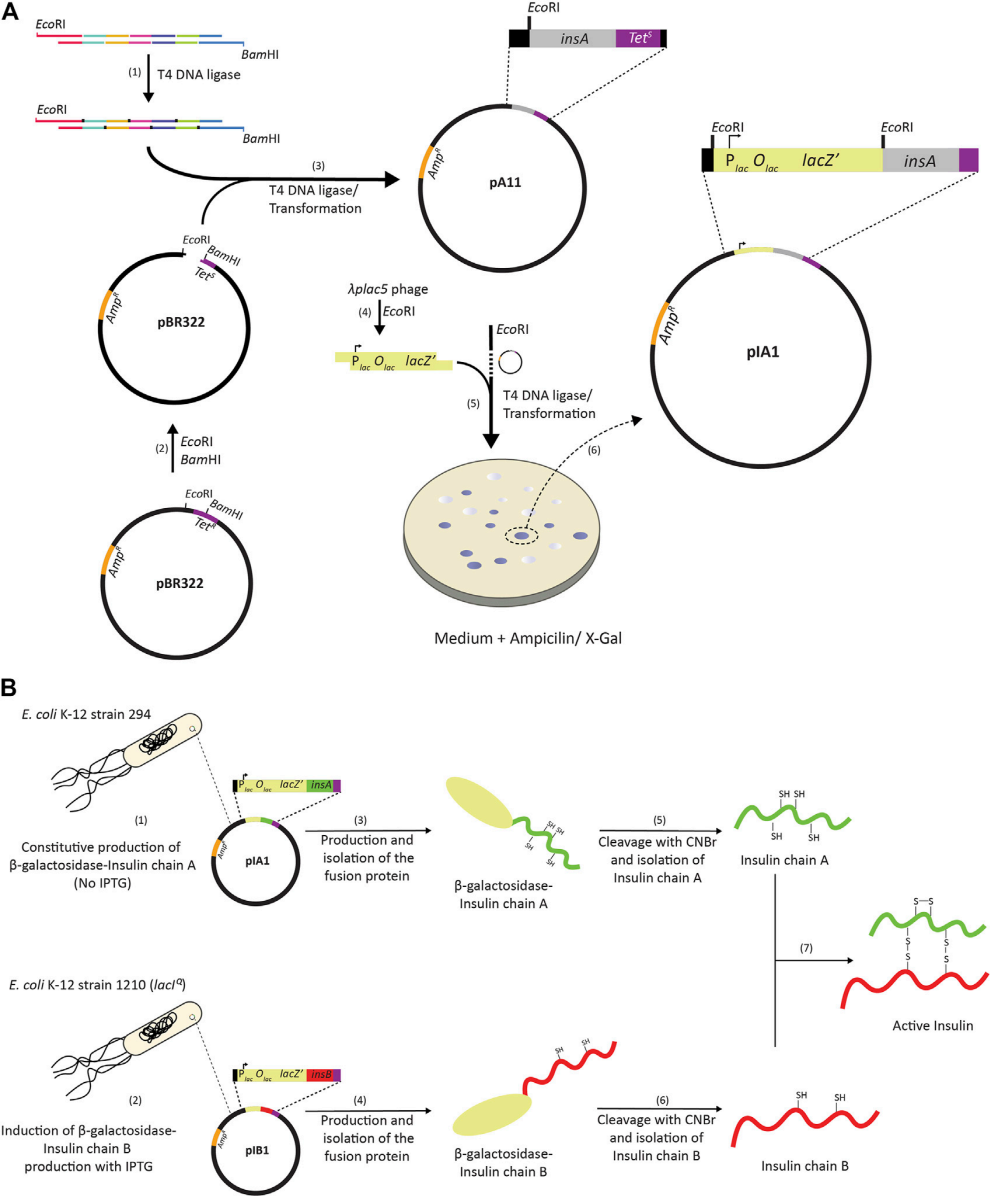
Cloning and production of human insulin using *E. coli*. **(A)**. The gene encoding the insulin A chain was constructed using DNA oligos with overlapping parts and T4 DNA ligase **(1)**. *Eco*RI and *Bam*HI compatible overhangs were introduced to the 5′ end and 3′ end of the *insA* gene, respectively. The overhangs were introduced to facilitate cloning of the *insA* gene into the pBR322 vector that had been digested with *Eco*RI and *Bam*HI **(2)**. After ligation and transformation **(3)**, positive clones were selected on antibiotic containing media; cells containing the pBR322 vector with the *insA* insert are ampicillin resistant, Amp^R^, and tetracycline sensitive, Tet^S^, since the insertion of *insA* into pBR322 results in the partial deletion of the *tet* gene. Insertion of the insulin chain gene into pBR322 was verified by DNA sequencing. Subsequently, an *Eco*RI fragment from the bacteriophage *λplac5* genome **(4)** containing the *lac* promoter, the *lac* operator and 1,006 codons of the *lacZ* gene (out of the 1,024 codons encoding full length β-galactosidase) was inserted into the *Eco*RI site of the pBR322 vector with the *insA* gene **(5)**. This results in the in-frame fusion of *lacZ* and *insA* enabling the production of a β-galactosidase insulin chain A fusion protein. Plates with medium containing ampicillin and the β-galactosidase substrate 5-bromo-4-chloro-3-indolyl-β-d-galactopyranoside (X-Gal) was used to identify blue colonies, *i.e*., colonies that could produce the β-galactosidase insulin A fusion protein **(6)**. An expression vector for the production of a β-galactosidase insulin chain B fusion was constructed in a similar way. **(B)** The A and B chains of insulin C-terminally fused to β-galactosidase were produced in the cytoplasm using two different *E. coli* strains. *E. coli* K-12 strain 294 was used for the production of the β-galactosidase-InsA fusion **(1)** and *E. coli* K-12 strain D1290 was used for the production of the β-galactosidase-InsB fusion **(2)**. *E. coli* K-12 strain 294 produces the β-galactosidase-InsA fusion constitutively, so no Isopropyl β-d-1-thiogalactopyranoside (IPTG) was used to induce the expression of the gene encoding the fusion. The *E. coli* K-12 strain D1290 contains a mutation in the promoter of the *lacI* gene (*lacI^Q^*), which results in higher production of LacI. Here, IPTG had to be added to induce expression of the gene encoding the fusion protein. After partial purification of the fusion proteins **(3, 4)**, the insulin chains were recovered from the fusion proteins by cyanogen treatment **(5, 6)**. Subsequently, the A and B chains were isolated and joined through air oxidation resulting in active insulin **(7)**.

In 1972, the creation of the first recombinant DNA molecules was reported (Jackson et al., 1972). Not long thereafter, a study describing the “Construction of Biologically Functional Bacterial Plasmids *In Vitro*” was published (Cohen et al., 1973). Together with the development of methodology to chemically synthesize DNA, these two studies paved the way for using *Escherichia coli* as a host for producing recombinant proteins (Itakura et al., 1977). In 1976, the company Genentech—its name is a play of words of “genetic engineering” and “technology”—was founded and it pioneered the use of *E. coli* for the production of therapeutic proteins (Hughes, 2011). The foundation of Genentech is widely considered as the start of recombinant DNA driven biotechnology. Based on a collaborative effort between Genentech and Eli Lilly, human insulin (a.k.a. humulin), became in 1982 the first recombinantly produced therapeutic protein approved by the FDA for human use (Crea et al., 1978; Goeddel et al., 1979). For the production of humulin, the A and B chains of insulin were produced in the cytoplasm of two different *E. coli* strains as C-terminal fusions to β-galactosidase (Figure 1). After partial purification of the chimeric proteins, the insulin chains were released from the fusion proteins by cyanogen treatment. Subsequently, the released A and B chains were purified and joined through air oxidation resulting in functional insulin.

First, recombinant proteins, like above mentioned β-galactosidase insulin fusion proteins, were produced in the cytoplasm of *E. coli* (*e.g.,* (Itakura et al., 1977; Goeddel et al., 1979)). Later on, based on increasing knowledge of protein export in *E. coli*, recombinant proteins were also produced in the periplasm of *E. coli* (*e.g.,* (Fraser and Bruce, 1978; Talmadge et al., 1980)). What are the (potential) benefits of producing a protein in the periplasm of *E. coli* rather than in its cytoplasm?

To reach the periplasm, a protein has to be equipped at its N-terminus with a cleavable signal peptide so that it can cross the cytoplasmic membrane (Hegde and Bernstein, 2006; Smets et al., 2019). Upon translocation across the membrane, the signal peptide is cleaved off. This enables to control which amino acid is at the very N-terminus of the mature protein (Smets et al., 2019). Not all proteins naturally contain a methionine at their N-terminus and the cytoplasmic production of a protein in the cytoplasm does not guarantee the presence of an N-terminal methionine due to the action of methionine aminopeptidase (Liao et al., 2004; Frottin et al., 2006). Removal of the N-formyl methionine can be critical for the proper folding, stability and function of a recombinant protein (Endo et al., 2001; Liao et al., 2003).

Protein production in the periplasm enables to isolate the protein from the periplasmic fraction rather than a whole cell lysate. This can greatly facilitate the isolation of a protein since it has to be isolated from a mixture that is less complex (Mergulhao et al., 2005). In this respect it should be mentioned that it is relatively easy to isolate the periplasmic fraction of *E. coli* also at an industrial scale (Mergulhao et al., 2005; Ellis and Humphreys, 2014). Furthermore, periplasmic protein production can also lead to the “spontaneous” release of the protein into the extracellular milieu (Kastenhofer et al., 2021). The release of a protein into the extracellular milieu can also be promoted by actively making the outer membrane more permeable or by fusing the protein to a fusion partner that is naturally secreted into the extracellular milieu (Hsiung et al., 1989; Zhang et al., 2006; Natarajan et al., 2017; Gao et al., 2018)). Extracellular production of a (fusion) protein can even further facilitate protein isolation (Tripathi, 2016). Notably, the costs involved in the isolation of a protein usually make up a large part of the total production costs (Tripathi and Shrivastava, 2019).

Many recombinant proteins, like hormones and antibody fragments, contain disulfide bonds which are essential for stabilizing the fold of a protein (Manta et al., 2019). Disulfide bond formation is not promoted in the reducing environment of the cytoplasm (Manta et al., 2019). However, the oxidative environment of the periplasm along with the presence of the disulfide bond formation (Dsb)-system in the periplasm promotes disulfide bond formation in this compartment, thereby facilitating the production of disulfide bond containing proteins (Manta et al., 2019). Finally, periplasmic protein production can promote the stability of a protein due to minimizing its exposure to cytoplasmic proteases (*e.g.,* (Talmadge and Gilbert, 1982)).

However, the production of a protein in the periplasm is often cumbersome. Usually, a significant fraction of the produced protein accumulates in the unprocessed form in the cytoplasm (Schlegel et al., 2013; Baumgarten et al., 2018; Karyolaimos et al., 2019; Karyolaimos et al., 2020). Periplasmic protein production can also have a negative effect on the fitness of the production host and consequently biomass formation as well as protein production yields (Schlegel et al., 2013; Baumgarten et al., 2018). Furthermore, proteins produced in the periplasm do not always fold properly and can also be prone to degradation (*e.g.,* (Meerman and Georgiou, 1994; Chen et al., 2004; Ellis et al., 2017)).

In this review, we give an overview of the different strategies used to promote proper targeting, efficient translocation, proper folding and avoid degradation of a protein when attempting to produce it in the periplasm of *E. coli*. We also discuss potential strategies to further enhance protein production yields in the periplasm.

## Export of Proteins From the Cytoplasm to the Periplasm

**FIGURE 2:**
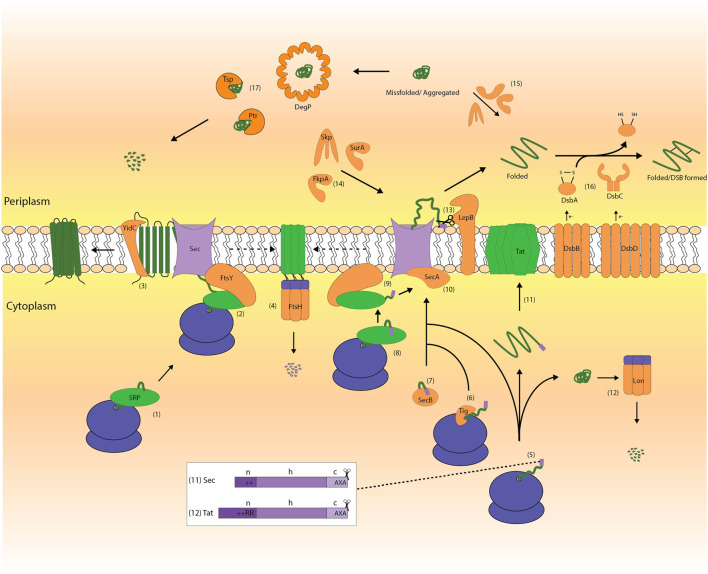
The biogenesis of membrane and secretory proteins in *E. coli*. Membrane and secretory proteins are synthesized in the cytoplasm by the ribosome **(1, 5)**. Most membrane proteins and some secretory proteins are targeted to the Sec-translocon *via* the SRP-pathway, which comprises the SRP and its receptor FtsY **(1–2, 8–9)**. The membrane protein YidC, which is an insertase/chaperone, can assist the biogenesis of membrane proteins in conjunction with the Sec-translocon as well as an independent entity **(3)**. Many secretory proteins are targeted post-translationally to the Sec-translocon in a chaperone-independent **(5, 10)** or -dependent manner **(6, 7, 10).** Chaperones like TF and SecB can facilitate post-translational protein targeting **(6, 7)**. FtsH degrades jammed Sec-translocons **(4)**. The TatABC-translocon mediates the translocation of folded proteins with a signal peptide containing the twin arginine motif **(11)**. Precursor proteins that get stuck in the cytoplasm can be degraded by cytoplasmic proteases like Lon **(12)**. The motor protein SecA pushes secretory proteins through the Sec-translocon channel **(10)**. Upon translocation of a protein across the cytoplasmic membrane LepB cleaves off the signal peptide **(13)**. Periplasmic folding modulators like SurA, Skp and FkpA catalyze the folding of proteins in the periplasm **(14, 15)**. In the periplasm, the Dsb-system, which consists of DsbA, B, C and D, mediates the formation of disulfide bonds **(16)** and proteins can be degraded by proteases like DegP, Tsp and Ptr **(17)**.

To set the stage for this review, we will first briefly summarize how proteins are exported from the cytoplasm to the periplasm in *E. coli*. A secretory protein is synthesized in the cytoplasm and has at its N-terminus a cleavable signal peptide that targets it to either the Sec-translocon or the Tat-translocon in the cytoplasmic membrane (Frain et al., 2019; Oswald et al., 2021) (Figure 2). In *E. coli*, the vast majority of secretory proteins is translocated across the cytoplasmic membrane *via* the Sec-translocon (Smets et al., 2019). During translocation *via* the Sec-translocon, secretory proteins are in an unfolded conformation (De Geyter et al., 2016). Proteins translocated across the cytoplasmic membrane *via* the Tat-translocon fold in the cytoplasm prior to their translocation across the cytoplasmic membrane (Frain et al., 2019; Palmer and Stansfeld, 2020). Upon their translocation the signal peptide is cleaved off from both Sec- and Tat-dependent proteins.

### Protein Targeting

The targeting of proteins to the Sec-translocon can occur either co-translationally *via* the Signal Recognition Particle (SRP)-pathway or post-translationally in a chaperone-dependent or -independent manner (Tsirigotaki et al., 2017; Smets et al., 2019) (Figure 2). The SRP-pathway comprises the signal recognition particle (SRP), which is a ribonucleoprotein particle consisting of the 4.5S RNA and the Ffh protein, and its receptor FtsY (Oswald et al., 2021). The cytoplasmic chaperones SecB and Trigger Factor (TF) can assist the post-translational targeting of proteins (Smets et al., 2019; De Geyter et al., 2020). SecB can bind to a subset of secretory proteins and keeps them in a translocation-competent state (Sala et al., 2014). TF, which plays a key role in the folding of cytoplasmic proteins, can also keep secretory proteins in a translocation-competent state and together with SecB aid their export (De Geyter et al., 2020). Targeting of (folded) proteins to the Tat-translocon is mediated by the presence of a signal peptide that contains a highly conserved twin-arginine motif (Sargent et al., 1998; Weiner et al., 1998; Frain et al., 2019; Palmer and Stansfeld, 2020).

### Protein Translocation

The core of the Sec-translocon consists of the membrane proteins SecY and SecE (Oswald et al., 2021) (Figure 2). Together they form a protein-conducting channel in the cytoplasmic membrane (Oswald et al., 2021). In the absence of SecE, SecY is degraded by the cytoplasmic membrane protease FtsH (Ito and Akiyama, 2005). The peripheral ATP-dependent motor protein SecA pushes secretory proteins through the Sec-translocon channel (Smets et al., 2019; Oswald et al., 2021). The mode of translocation through the Sec-translocon can affect how a protein folds in the periplasm (Kadokura and Beckwith, 2009). The membrane protein YidC, which is an insertase/chaperone, can assist the biogenesis of membrane proteins in conjunction with the Sec-translocon as well as an independent entity (Shanmugaw and Dalbey, 2019). The *E. coli* Tat-translocon comprises multiple copies of the membrane proteins TatA, TatB and TatC (Sargent et al., 1998; Weiner et al., 1998; Frain et al., 2019). The assembly of the Tat-translocon is dynamic and is triggered by a Tat substrate interacting through its signal peptide with the TatBC-receptor complex (Frain et al., 2019). The Tat-translocon has a quality-control mechanism that prevents translocation of not properly folded proteins (Frain et al., 2019).

Notably, the protein flux through the Tat-translocon is considerably lower than through the Sec-translocon. It takes a few minutes to translocate a Tat-dependent protein *versus* a few seconds for a Sec-dependent protein (Taw et al., 2021). The proton-motive force is required for both efficient Sec- and Tat-mediated protein export (Frain et al., 2019; Oswald et al., 2021).

### Protein Folding and Degradation

Upon translocation of a protein across the cytoplasmic membrane, signal peptidase I (LepB) cleaves off the signal peptide (Paetzel, 2014) (Figure 2). In the periplasm there are folding modulators that can assist the folding of Sec-dependent proteins (Stull et al., 2018). Proteins that require disulfide bonds are reduced when they are exported from the cytoplasm to the periplasm *via* the Sec-translocon (De Geyter et al., 2016; Smets et al., 2019). In the periplasm, the Dsb-system, which consists of DsbA, B, C and D, mediates the formation of disulfide bonds (Manta et al., 2019). Oxidized DsbA transfers its own disulfide bond to its substrate proteins and is reduced in the process. The membrane protein DsbB re-oxidizes DsbA and it donates the electrons it has received from DsbA to the respiratory chain. DsbC can recognize if the substrate protein is not properly oxidized by DsbA. DsbC either isomerizes substrates that are not properly oxidized to their native state or reduces them thereby allowing DsbA to have another go to introduce a proper disulfide bond. The membrane protein DsbD maintains DsbC in its active reduced state. DsbD receives its electrons from the cytoplasmic thioredoxin TrxA, which receives its electrons from NADPH. Other examples of periplasmic folding modulators are FkpA, which is a periplasmic chaperone/peptidyl-prolyl isomerase (PPIase), SurA, which is a chaperone/PPIase, and the chaperone Skp (Stull et al., 2018). It should be noted that our grasp of the repertoire of periplasmic folding modulators and folding modulator–substrate specificity is still rather poor (Stull et al., 2018). The same holds for periplasmic proteases and periplasmic protease–substrate specificity (Merdanovic et al., 2011).

### Signal Peptides

A signal peptide consists of a positively charged N-terminal region (n-region), an apolar hydrophobic core (h-region) and a more polar C-terminal region (c-region) (Izard and Kendall, 1994; Tsirigotaki et al., 2017). The c-region contains an AXA motif (Paetzel, 2014). This motif is recognized by LepB, which cleaves off the signal peptide upon protein translocation across the membrane (Paetzel, 2014). As mentioned before, Tat-dependent signal peptides contain a conserved twin arginine motif in the n-region, at the boundary with the h-region (Frain et al., 2019). The hydrophobicity of the h-region of signal peptides that guide secretory protein to the Sec-translocon plays an important role in the mode of targeting (*e.g.,* (Rusch and Kendall, 1994; Chen et al., 1996; Rusch et al., 2002)). It has been shown that increasing the hydrophobicity of the h-region of a signal peptide can funnel proteins that are normally targeted post-translationally into the co-translational SRP-pathway and *vice versa* (Kim et al., 2001). Furthermore, it has been shown that targeting of an export-defective protein can be rescued by increasing the hydrophobicity of the h-region (Rusch and Kendall, 1994). By funneling the protein into the co-translational SRP-pathway the premature folding of the protein in the cytoplasm is most likely prevented (Rusch and Kendall, 1994). Signal peptides of post-translationally targeted proteins can help to delay the folding of the mature domain in the cytoplasm (Tsirigotaki et al., 2018). Recently, it has been shown that post-translational targeting/translocation is not only mediated by the signal peptide, but also by the so-called mature domain targeting sites in the mature part of the preprotein that can also bind to SecA (Chatzi et al., 2017; Smets et al., 2019)**.**

## Strategies to Enhance Periplasmic Protein Production Yields

Strategies to enhance periplasmic protein production yields have focused on harmonizing protein production rates with the capacity of the secretory apparatus by transcriptional and translational tuning, by signal peptide selection and engineering, by increasing the targeting, translocation and periplasmic folding capacity of the cell, by preventing proteolysis, and by natural and engineered adaptation of the production host. Using notable examples as a thread, we will go through the different strategies used to enhance periplasmic protein production yields. Importantly, if the reader contemplates to use a certain strategy to enhance periplasmic protein production yields, the technical setup used to implement the strategy should be carefully considered.

### Tuning Transcription and Translation

Studies aiming to enhance membrane protein production yields in *E. coli* have played a key role in identifying what can hamper periplasmic protein production. Therefore, first a brief introduction to these studies, in which the BL21(DE3) strain and T7 promoter-based expression vectors were used for protein production, will be given (Studier and Moffatt, 1986). In BL21(DE3), the expression of the gene encoding the target protein is driven by the bacteriophage T7 RNA polymerase (Studier and Moffatt, 1986). This polymerase transcribes approximately 5 times faster than *E. coli* RNA polymerase (Chamberlin et al., 1970; Studier and Moffatt, 1986). The T7 RNA polymerase specifically recognizes the T7 promoter governing the expression of the gene encoding the target protein (Studier and Moffatt, 1986). Expression of the chromosomally localized gene encoding the T7 RNA polymerase is governed by the IPTG-inducible *lac*UV5 promoter (Studier and Moffatt, 1986). This promoter is a more powerful variant of the wild-type *lac* promoter and it is not well titratable (Silverstone et al., 1970; Wanner et al., 1977). The idea behind this setup is that the more mRNA encoding the recombinant protein is synthesized, the more protein can be produced. In this respect it is good to mention that the system was developed for the production of soluble proteins in the cytoplasm (Studier and Moffatt, 1986).

The over-production of membrane proteins in BL21(DE3) is usually toxic (Wagner et al., 2007). However, many membrane proteins can be efficiently produced in the BL21(DE3)-derived, C41(DE3) and C43(DE3) strains (Miroux and Walker, 1996). Based on the observation that in the C41(DE3) and C43(DE3) strains the promoter governing the expression of *t7rnap* is weakened, the BL21(DE3)-derived Lemo21(DE3) strain was constructed (Wagner et al., 2008). In this strain, the activity of the T7 RNA polymerase can be precisely controlled by its natural inhibitor T7 lysozyme (Moffatt and Studier, 1987). The gene encoding the T7 lysozyme is on a plasmid under control of a rhamnose promoter. This promoter is titratable and covers a broad range of expression intensities (Giacalone et al., 2006). The combination of the *lac*UV5 promoter governing the expression of the gene encoding the T7 RNA polymerase and the rhamnose promoter governing the expression of the gene encoding T7 lysozyme, enables to tune the expression intensity of the gene encoding the target membrane protein from a T7 promoter-based expression vector (Wagner et al., 2008). As a consequence, the membrane protein production rate can be harmonized with the capacity of the membrane protein biogenesis apparatus of the cell using transcriptional tuning (Wagner et al., 2008; Schlegel et al., 2012). This results in cells in which the capacity of membrane protein biogenesis apparatus is not saturated. Therefore, these cells can efficiently produce membrane proteins (Wagner et al., 2008; Schlegel et al., 2012).

The Lemo21(DE3) strain was also used to test if saturation of the secretory apparatus can cause problems when attempting to produce proteins in the periplasm (Schlegel et al., 2013). Indeed, periplasmic protein production can also lead to saturation of the secretory apparatus. This results in poor periplasmic protein production yields and accumulation of the precursor target protein in the cytoplasm, poor growth, precursor accumulation of endogenous secretory proteins in the cytoplasm and the induction of the heat shock response, which is indicative for protein misfolding and mistargeting (Schlegel et al., 2013). Harmonizing the production rate of a secretory protein with the capacity of the secretory apparatus leads to more protein produced in the periplasm and strongly reduced negative effects (Schlegel et al., 2013). When the proteome composition of Lemo21(DE3) cells producing a single chain antibody fragment (scFv) under optimized conditions was compared with the proteome composition of Lemo21(DE3) cells with an empty expression vector, there were no notable differences (Baumgarten et al., 2018). Thus, the scFv could be efficiently produced in the periplasm without causing any notable stress or other side effects.

The rhamnose inducible promoter can also be used to directly express a gene encoding a protein (Giacalone et al., 2006; Hjelm et al., 2017). When the rhamnose promoter-mediated production of (cytoplasmic) super folder GFP was monitored in real-time using different amounts of rhamnose in *E. coli* wild-type cells, it became clear that the rhamnose concentration-dependent tunability is due to rhamnose consumption rather than regulating protein production rates (Hjelm et al., 2017). To regulate rhamnose promoter-mediated protein production rates in a rhamnose concentration dependent manner, a strain background in which both rhamnose catabolism as well as rhamnose uptake by the RhaT-transporter are abolished has to be used (Hjelm et al., 2017). Unfortunately, there is no straight forward explanation for this observation. Thus, using the rhamnose promoter in a rhamnose catabolism and *rhaT* deficient strain background enables harmonizing target protein production rates with the capacity of the secretory apparatus in a rhamnose concentration dependent manner (Hjelm et al., 2017). This setup has been used successfully to boost periplasmic protein production yields (Hjelm et al., 2017; Karyolaimos et al., 2019).

**FIGURE 3:**
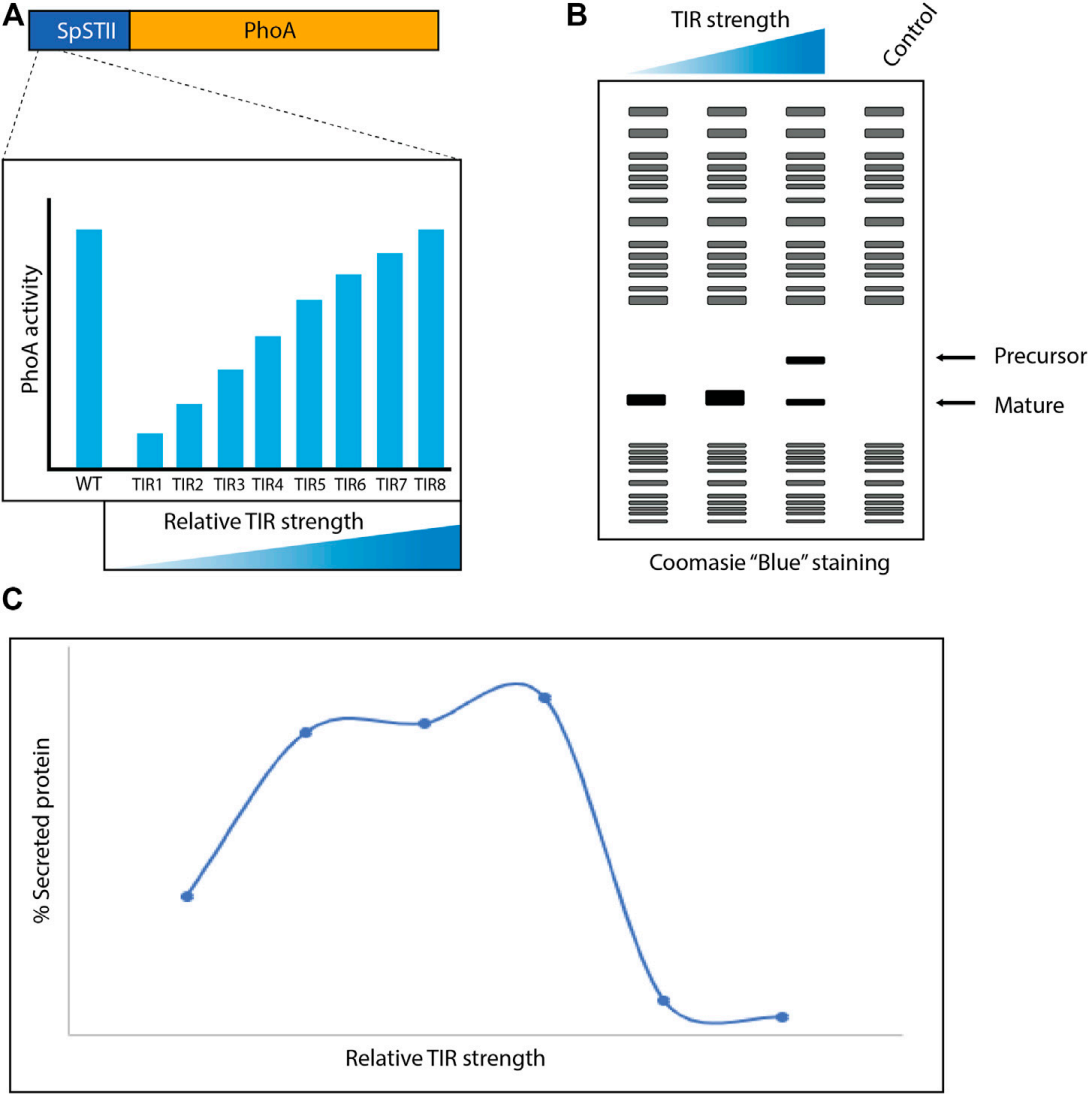
Modifying the translational initiation region (TIR) to enhance recombinant protein production in the periplasm. (**A)** Codons 2 to 6 of the STII signal peptide were modified without changing the amino acid sequence of the signal peptide. The relative TIR strength of variants was assessed using PhoA as a reporter and TIR variants covering a 10-fold range of translational strength were identified. **(B)** Production of recombinant protein targets was tuned using the TIR library and protein production was assessed using SDS-PAGE and Coomassie Blue staining. This led to the identification of a TIR optimal for the production of a particular recombinant protein in the periplasm. **(C)** Mid-range relative TIR strengths lead in general to the highest periplasmic protein production yields.

Researchers from Genentech reported in 1996 that the secretion of recombinant proteins into the periplasm of *E. coli* could be enhanced by optimizing translational levels rather than maximizing them (Simmons and Yansura, 1996) (Figure 3). The signal peptide from the *E. coli* heat-stable enterotoxin II (STII) in combination with the phosphate limitation inducible *phoA* promoter was used to produce a series of recombinant proteins in the periplasm (Chang et al., 1987; Simmons and Yansura, 1996). Notably, STII is targeted post-translationally to the Sec-translocon. To tune translational levels, the translational initiation region (TIR), which covers the immediate upstream part of the ribosome binding site to approximately 20 nucleotides downstream of the initiation codon, was modified (Goodman et al., 2013). More precisely, codons 2 to 6 of the STII signal peptide were modified without changing the amino acid sequence of the signal peptide to create a library of vectors with different translational strengths. Subsequently, the *E. coli* alkaline phosphatase (PhoA) was used as a reporter to characterize the library. This enabled to identify TIR variants covering a 10-fold range of translational strength. These variants were used to enhance periplasmic protein production. Importantly, for each target tested a narrow translational range was required for optimal periplasmic protein production. Interestingly, when an optimal TIR had been identified for a target and the copy number of the expression vector used was increased, periplasmic protein production levels were strongly reduced and precursor material accumulated in the cytoplasm. At the time the authors could only speculate about the underlying mechanisms behind their findings. However, based on what we know now it is obvious that using different TIRs the production rate of a secretory recombinant protein can be harmonized with the capacity of the secretory apparatus, thereby enhancing periplasmic production yields. Increasing the copy number of the expression vector leads to increased protein production and, consequently, the detrimental saturation of the secretory apparatus (Schlegel et al., 2013; Baumgarten et al., 2018).

Taken together, harmonizing secretory protein production rates with the capacity of the secretory apparatus by transcriptional and translational tuning can lead to enhanced periplasmic protein production yields. To regulate secretory protein production rates at the transcriptional level, titratable promoter systems other than the ones described above can also be used (Meyer et al., 2019). To regulate secretory protein production rates at the translational level the ribosome binding site of a TIR can also be modified (Salis et al., 2009; Eichmann et al., 2019). Rather than using TIR libraries, screening approaches can also be used to identify a suitable TIR (Rennig et al., 2018). The T7 RNA polymerase-based RiboTite system, which was recently used to enhance protein production yields in the periplasm, is an example of a system that operates both at the transcriptional and translational level (Horga et al., 2018). In the RiboTite system, transcriptional control is mediated by an IPTG-inducible promoter controlling *t7rnap* expression (Morra et al., 2016). Translational control in the system is mediated by an orthogonal riboswitch (Morra et al., 2016). This switch sequesters the ribosome binding site in the absence and releases it in the presence of pyrimido-pyrimidine-2,4-diamine.

### Signal Peptide Selection and Engineering

With our current knowledge it is not possible to predict what signal peptide one should use to efficiently produce a protein in the periplasm of *E. coli* (Karyolaimos et al., 2019). Often people use signal peptides from abundantly present native proteins or signal peptides that have successfully been used before for periplasmic protein production (Kendall et al., 1986; Schierle et al., 2003; Sijbrandi et al., 2003; Schlegel et al., 2013; Schmidt et al., 2016; Hjelm et al., 2017; Baumgarten et al., 2018; Karyolaimos et al., 2019). However, there is no guarantee that these signal peptides will also facilitate the efficient production of any other protein in the periplasm (Karyolaimos et al., 2019). Systematic screening or engineering approaches to identify signal peptides that enhance periplasmic protein production yields are scarce.

To improve the display levels of a series of proteins on filamentous phages, the Plückthun laboratory used a panel of signal peptides that promote co-translational as well as post-translational targeting of phage coat protein fusions to the Sec-translocon (Steiner et al., 2006). Display levels of the proteins of interest were markedly higher when signal peptides promoting co-translational targeting were used and for some targets levels could be enhanced up to seven hundred fold. Co-translational targeting of the fusions may prevent folding of the protein of interest moieties in the cytoplasm, which would prevent translocation and the subsequent display of the fusions.

**FIGURE 4:**
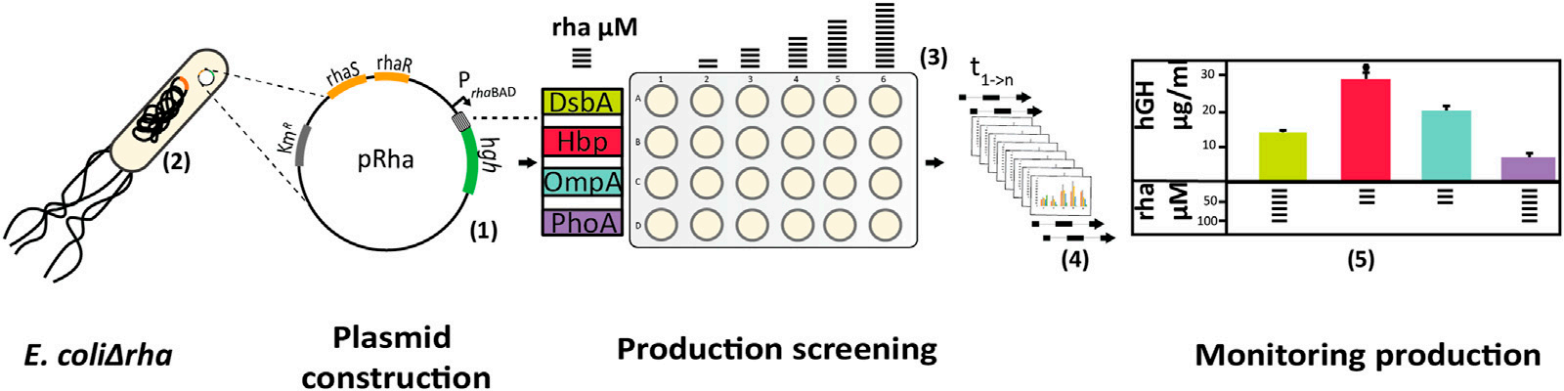
Setup of the signal peptide and production rate-based combinatorial screening approach to enhance periplasmic protein production yields in *E. coli*. The gene encoding the recombinant protein to be produced (hGH) was fused to sequences encoding for the signal peptides from the *E. coli* proteins DsbA, OmpA, PhoA, and the Hbp. The genetic fusions were subsequently inserted into the rhamnose promoter-based expression vector pRha **(1)**. The vectors were transformed into *E. coliΔrha* **(2)**. In *E. coliΔrha*, target production rates can be precisely tuned by varying the rhamnose concentration **(3)**. Production screening of the periplasmically produced hGH was done in a 24-well setup and a range of rhamnose concentrations was used to induce the target gene expression **(3)**. Following the induction, the cells from equal culture volumes were harvested at different time intervals and hGH production was monitored by means of fluorescent western blotting **(4)**. The best conditions for each signal peptide combination were compared directly to determine the rhamnose concentration and signal peptide combination that provided the highest periplasmic yields of active hGH **(5)**.

Importantly, not only signal peptides can significantly impact periplasmic protein production yields, but as discussed in the previous section also protein production rates. Therefore, our lab set up a combined screen involving four different signal peptides and varying production rates to produce a scFv and human growth hormone (hGH) in the periplasm (Karyolaimos et al., 2019) (Figure 4). The signal peptides from the *E. coli* DsbA, Hbp, OmpA and PhoA proteins were used to guide the two targets to the periplasm. DsbA is, as explained before**,** a periplasmic thiol:disulfide oxidoreductase and it is generally assumed that the DsbA signal peptide mediates the co-translational targeting of proteins (Schierle et al., 2003). The DsbA signal peptide has been widely and successfully used for the production of recombinant proteins in the periplasm (*e.g.,* (Steiner et al., 2006)). The Hemoglobin protease (Hbp) is an abundantly produced autotransporter and is co-translationally targeted (Sijbrandi et al., 2003). OmpA is an outer membrane protein and targeted post-translationally in a SecB-dependent manner (Baars et al., 2006). PhoA is a periplasmic enzyme that catalyses the hydrolysis and transphosphorylation of a wide variety of phosphate monoesters and it has been reported that PhoA targeting can occur both post- and co-translationally (Kendall et al., 1986; Kadokura and Beckwith, 2009). The previously discussed rhamnose promoter (see section “Tuning transcription and translation”) and a strain background in which the whole *rha* operon is deleted was used to produce the scFv and hGH equipped with the four different signal peptides at different production rates. Across the screen conditions, the periplasmic production yields of both targets varied a lot. The optimal signal peptide and rhamnose concentration differed for each protein. Interestingly, for scFv the OmpA signal and for hGH the Hbp signal peptide led to the best periplasmic production yields. Thus, the signal peptide from the post-translationally targeted OmpA protein and the signal peptide from a co-translationally targeted protein Hbp protein result in optimal periplasmic production of the scFv and hGH, respectively. There is no straightforward explanation for this observation, but it stresses that signal peptides derived from post-translationally targeted proteins cannot be excluded *a priori* for efficient periplasmic protein production. This study also shows the importance of combinatorial screening approaches for enhancing periplasmic protein production yields.

Rather than trying to identify a suitable signal peptide for the periplasmic production of a protein in the periplasm, such a signal peptide can also be engineered. In a study from Genentech, signal peptide engineering was used to promote the secretion of the heavy chain of a monoclonal antibody (mAb) that was limiting the production of the mAb in the periplasm (Zhou et al., 2016). To improve the secretion of the heavy chain it was produced using different signal peptides at controlled TIR strengths. The use of the DsbA signal peptide resulted in the highest periplasmic production yields compared to the other signal peptides tested. A close inspection of the different signal peptides used showed that the hydrophobicity of the h-region of the DsbA signal peptide was higher than the hydrophobicity of the h-region of the other signal peptides. This raised the question if the hydrophobicity of the h-region of a signal peptide can play an important role in periplasmic protein production. To explore this the signal peptide of the aforementioned STII protein was used since its n- and c-regions are very similar to the same regions of the DsbA signal peptide. The hydrophobicity of the h-region of the STII signal peptide is lower and increasing the hydrophobicity of its h-region enhanced secretion of the heavy chain and periplasmic mAb production levels. Further increasing the hydrophobicity of the h-region did not result in further enhancing periplasmic production yields. Decreasing the hydrophobicity of the h-region of the DsbA signal resulted in the opposite effect. Thus, in this study promoting co-translational targeting led to enhanced periplasmic protein production.

In several studies it has been shown that the presence of rare codons in the genetic information encoding a signal peptide, especially in the region encoding the n-region, can have a positive impact on both periplasmic protein production yields and on proper protein folding (*e.g.,* (Humphreys et al., 2000; Zalucki et al., 2008; Zalucki et al., 2010; Castiñeiras et al., 2018; Kulmala et al., 2019)). It is tempting to speculate that these rare codons make the TIR/protein translocation kinetics more amenable to efficient periplasmic protein production.

### Increasing the Capacity of the Secretory Apparatus

#### Increasing Protein Targeting Capacity

Co-producing the cytoplasmic chaperones GroEL and DnaK, which both have also been implicated in protein secretion, can facilitate periplasmic protein production (Hu et al., 2007; Makino et al., 2011b; Sonoda et al., 2011). Attempts to enhance periplasmic protein production yields by co-producing TF and SecB have not been successful (Puertas et al., 2010; Makino et al., 2011b). TF can hamper protein secretion by blocking access of the precursor protein to the secretory apparatus (De Geyter et al., 2020). It is possible that increased levels of SecB hamper secretion by retaining a secretory protein in the cytoplasm rather than promoting its secretion. Interestingly, the production of the secreted form of a leech carboxypeptidase inhibitor, targeted by the DsbA signal peptide to the Sec-translocon, is increased in a TF deficient strain background (Puertas et al., 2010). It is possible that in spite of using the DsbA signal peptide, targeting of the leech carboxypeptidase inhibitor occurs only partially co-translationally due to limited SRP-pathway capacity. If this would be the case, the secretion of the target protein could indeed be hampered by TF. This scenario is supported by the observations that co-producing SRP (both Ffh as well as the 4.5S RNA) also increased production of the leech carboxypeptidase inhibitor. Moreover, combining a TF deficient strain background with the co-production of SRP had an additive effect on the production of secreted target protein and strongly reduced the accumulation of the precursor form of the target protein in the cytoplasm (Puertas et al., 2010). Co-production of components of the SRP-targeting pathway, including FtsY, have been widely used to facilitate the co-translational targeting of secretory recombinant proteins (*e.g.,* (Lee et al., 2013)).

#### Increasing Protein Translocation Capacity

It has been shown that co-production of the Sec-translocon core components SecY and E can lead to increased periplasmic protein production yields (Makino et al., 2011b). However, when SecY and E were co-produced to increase periplasmic production of human interleukin-6 equipped with an OmpA signal peptide, periplasmic production yields were not increased (Pérez-Pérez et al., 1994). Interestingly, when a mutant version of SecY, *i.e*., PrlA4, was co-produced rather than wild-type SecY, the periplasmic production yield of human interleukin-6 increased significantly. PrlA4 was isolated in a screen aiming at the isolation of suppressor mutations that could restore the export of LamB equipped with defective signal peptides (Emr et al., 1981). Co-production of PrlA4 without SecE provided a three-fold increase in export of the target protein compared to the 10-fold increase when both SecE and PrlA4 were co-produced. Co-production of SecE with PrlA4 is most likely needed to prevent SecY/PrlA4 degradation by FtsH (Nijeholt et al., 2013). PrlA4 has not only been shown to enable the translocation of proteins with a defective signal peptide or without a signal peptide, but it also has been shown to have a higher binding affinity towards SecA (van der Wolk et al., 1998). This promotes binding of SecA as well as the precursor protein at the translocation site. This may improve the translocation efficiency of proteins the wild-type Sec-translocon has difficulties with translocating (van der Wolk et al., 1998).

The Tat pathway has also been used to produce proteins, including ones with disulfide bonds, in the periplasm of *E. coli* (*e.g.,* (DeLisa et al., 2003; Matos et al., 2014; Alanen et al., 2015; Browning et al., 2017; Montero et al., 2019)). Co-producing the components of the Tat-translocon has been used successfully to increase the Tat-pathway capacity of *E. coli*, thereby enhancing Tat-mediated periplasmic protein production yields (Browning et al., 2017; Montero et al., 2019). To enhance the periplasmic production of properly folded disulfide bond containing proteins in *E. coli*, co-producing Tat-translocon components has been combined with the CyDisCo (Cytoplasmic Disulfide bond formation in *E. coli*) setup. The CyDisCo setup is based on the cytoplasmic production of a yeast mitochondrial thiol oxidase, Erv1p, and the human protein disulfide isomerase, PDI, both lacking signal peptides (Gaciarz et al., 2016).

Recently, Tat-translocons were isolated enabling high level periplasmic protein production (Taw et al., 2021). To isolate these translocons, cells with the native *tat* operon deleted and expressing a mutant library of *tatABC* operons and at the same time producing a scFv equipped with a Tat-dependent signal peptide and C-terminally fused to TEM-β lactamase were put on plates containing a high amount of carbenicillin. This setup enabled to use growth for selecting cells with an increased Tat-export capacity and resulted in the isolation of three “super-secreting” Tat-translocons. These translocons not only mediated the efficient periplasmic production of the target used in the screen, but also of other proteins at levels that are higher than the ones obtained with the wild-type Tat-translocon. All three “super-secreting” Tat-translocons had mutations in the *tatB* and *tatC* genes and no mutations in the *tatA* gene. Using targets that are rejected by the wild-type Tat-translocon it was shown that the quality-control mechanism that prevents translocation of not properly folded proteins in the mutant Tat-translocons appears to be more relaxed. This most likely enables an increased protein flux through these translocons. Importantly, this does not affect the quality of the in the periplasm produced proteins.

#### Increasing Signal Peptide Processing Capacity

Co-producing LepB, which cleaves off signal peptides upon protein translocation, can also increase periplasmic protein production yields (van Dijl et al., 1991). It has been shown that increasing the synthesis of protease IV (SppA) by modifying its TIR also results in enhanced periplasmic protein production (Gawin et al., 2020). SppA is a membrane-bound signal peptide peptidase that is required for maintaining proper protein secretion (Novak and Dev, 1988; Dalbey et al., 2012).

#### Increasing Periplasmic Protein Folding Capacity

Periplasmic folding modulators have also been co-produced to enhance periplasmic protein production yields. Co-production of one of the or both key players of the Dsb-system, *i.e*., DsbA and DsbC, has been widely used to enhance the production of disulfide bond containing proteins in the periplasm (*e.g.,* (Joly et al., 1998; Schlapschy et al., 2006; Makino et al., 2011b; Sonoda et al., 2011)). Co-production of *e.g*., FkpA, SurA and Skp has also helped to enhance periplasmic and extracellular protein production yields (*e.g.,* (Mavrangelos et al., 2001; Schlapschy et al., 2006; Hu et al., 2007; Makino et al., 2011a; Sonoda et al., 2011)). To facilitate the identification of folding modulators enhancing periplasmic protein production yields, expression vectors that enable the co-production of several folding modulators at the same time have been engineered (Schlapschy et al., 2006). It has been shown that culturing setups can determine what folding modulator has to be co-produced (Ellis et al., 2017). To enhance periplasmic production of an antibody fragment in small shake flasks FkpA had to be co-produced. However, in a high cell density fed-batch culture DsbC had to be co-produced. This observation raises the question if the co-production of a folding modulator can also have indirect positive effects. Indeed, it has been shown that co-producing folding modulators can have a positive effect on the fitness of *E. coli*, which also can help to enhance periplasmic protein production yields (Ow et al., 2010).

Most often two compatible expression vectors have been used for producing the folding modulator and the recombinant protein (*e.g.,* (Joly et al., 1998; Schlapschy et al., 2006; Makino et al., 2011b; Sonoda et al., 2011)). Usually, different promoter systems are used to govern the expression of the genes encoding the folding modulator and the protein. The different promoter systems can also be used for the sequential production of a folding modulator and the protein (Joly et al., 1998; Makino et al., 2011b). Thus, cells can be pre-loaded with a folding modulator before the production of the protein is initiated. This better prepares cells for the production of the protein and lowers the risk of saturating the secretory apparatus. Also artificial operons containing the genes encoding both the folding modulator and the protein have been used (Ellis et al., 2017). This setup requires only one expression vector and one promoter system and is in particular useful for large scale protein production.

### Preventing Proteolysis

An important reason to produce a protein in the periplasm is to minimize its exposure to cytoplasmic proteases (Talmadge and Gilbert, 1982). However, this does not mean that there are no proteases in the cell envelope which can negatively affect periplasmic protein production (Gottesman, 1996). Indeed, it has been shown that the three periplasmic proteases DegP, Tsp and Ptr, can negatively affect protein production yields in the periplasm (Meerman and Georgiou, 1994; Chen et al., 2004). Most work on the identification of proteases affecting periplasmic protein production yields was done at a time when these proteases were the main ones that had been characterized (Gottesman, 1996). Only little is known about their substrates and how they recognize them. Therefore, it cannot be predicted which protease or combination of proteases can negatively affect production yields of a particular protein in the periplasm. This makes that proteases interfering with periplasmic protein production have to be identified by screening protein production yields in protease deficient strains.

The serine endoprotease DegP was identified in a screen aiming at the isolation of mutations preventing the degradation of abnormal periplasmic proteins (Strauch and Beckwith, 1988). DegP may degrade transiently denatured, unfolded proteins and/or newly secreted proteins prior to folding and disulfide bond formation (Strauch et al., 1989; Laskowska et al., 1996; Skorko-Glonek et al., 1999; Soltes et al., 2017). Inactivation of DegP by deleting the *degP* gene often results in improved periplasmic protein production yields (*e.g*., (Meerman and Georgiou, 1994; Champion et al., 2001; Chen et al., 2004)). DegP can also act as a chaperone (Meltzer et al., 2009). The protease function of DegP can be inactivated by a simple point mutation without affecting its chaperone activity (Spiess et al., 1999). This mutation is often used to create DegP protease deficient strains that still have DegP chaperone activity (*e.g.,* (Ellis et al., 2017)). Rather than deleting the gene encoding DegP or inactivating its protease function, periplasmic protein production yields have also been improved by lowering DegP levels. This was done by replacing the ribosome binding site in the TIR controlling *degP* expression with a weaker one (Gawin et al., 2020).

Both Tsp and Ptr were first detected when attempting to identify cytoplasmic proteases suggesting that these two periplasmic proteases are abundantly present (Cheng et al., 1979; Swamy and Goldberg, 1981). Tsp can degrade proteins with nonpolar C-termini and based on this observation it was named Tail-specific protease (Keiler and Sauer, 1996). It has been reported that Tsp can degrade antibody light chain fragments (Chen et al., 2004; Ellis et al., 2017). Tsp is also involved in regulating peptidoglycan synthesis by degrading MepS, which is a murein endopeptidase participating in the expansion of the peptidoglycan sacculus during growth and morphogenesis (Singh et al., 2015). Deletion of the gene encoding Tsp impairs growth. Inactivating the *mepS* gene in a Tsp deficient background restores growth (Chen et al., 2004; Ellis et al., 2017). Ptr (Protease III) is a zinc metalloendopeptidase that has been shown to degrade insulin (Swamy and Goldberg, 1981; Becker and Roth, 1992; Ding et al., 1992).

OmpT is an outer membrane protease with specificity for paired basic residues and its catalytic site is facing the bacterial surface (Kramer et al., 2001). OmpT can degrade periplasmically produced proteins after cell lysis (Grodberg and Dunn, 1988; Akiyama and Ito, 1990). Since proteins produced in the periplasm are usually isolated or can leak in the extracellular medium often OmpT deficient production strains are used (Jeong et al., 2009; Studier et al., 2009; Jeong et al., 2015).

Proteases do not only negatively affect periplasmic protein production yields, they can also be required for efficient periplasmic protein production. Secretory proteins can get stuck in the Sec-translocon thereby jamming it (Jiang et al., 2021). The cytoplasmic membrane protease FtsH, which is a processive, ATP-dependent zinc metallopeptidase for both cytoplasmic and cytoplasmic membrane proteins, can clear jammed Sec-translocons (van Stelten et al., 2009). Furthermore, it has been shown that the cytoplasmic Lon protease can clear not properly targeted secretory proteins in the cytoplasm (Kihara et al., 1995; van Stelten et al., 2009). It has been proposed that the cytoplasmic peptidase, PrlC (oligopeptidase A) can remove signal peptides from not properly targeted secretory proteins, thereby setting the stage for their clearance (Conlin et al., 1992).

### Natural and Engineered Adaptation of the Production Host

*E. coli* can respond to stress by changing its proteome composition (*e.g.,* (Wagner et al., 2007; Baumgarten et al., 2018)). It has been shown that if secretory protein production results in saturation of the secretory apparatus the heat shock response is activated to clear mis-targeted and -folded proteins and increase the protein (re)folding capacity of the cell (*e.g.,* (Wagner et al., 2007; Baumgarten et al., 2018)). Using the Lemo21(DE3) strain it has been shown that harmonizing the production rate of a secretory protein with the capacity of the secretory apparatus can result in cells that have a proteome composition that is similar to the proteome composition of non-producing cells (Baumgarten et al., 2018).

In the section “Tuning transcription and translation”, it was described how hGH was produced using the rhamnose promoter in a *rha* operon deletion strain background at varying concentrations of rhamnose and four different signal peptides. Cells producing hGH in the periplasm at the highest level, for each of the four signal peptides used, were analyzed using proteomics (Karyolaimos et al., 2020). Irrespective of the signal peptide used, the accumulation levels of SecA, LepB and YidC were increased. Thus, enhancing periplasmic hGH production using the rhamnose promoter in a *rha* operon deletion strain background leads to increased Sec-translocon capacity, increased capacity to cleave signal peptides from secretory proteins and an increased capacity of an alternative membrane protein biogenesis pathway, thereby freeing up Sec-translocon capacity for protein secretion. When cells with enhanced periplasmic hGH production yields were harvested and subsequently cultured in the absence of inducer, SecA, LepB, and YidC accumulation levels went down again. This indicates that *E. coli* can adapt its protein secretory apparatus for enhanced recombinant protein production in the periplasm. The gene encoding SecA is in an operon preceded by the gene encoding SecM, which is a secretion monitor (Ito et al., 2018). SecM is encoded by the 5′ region of the *secM-secA* mRNA. SecM translation is subject to transient elongation arrest, which is prolonged when the secretion of the nascent SecM is hampered. This results in disrupting the secondary structure of the *secM-secA* mRNA by the stalled ribosome such that the ribosome binding site of *secA* becomes available leading to the synthesis of SecA to relieve the export defect. Unfortunately, we have only limited knowledge on how LepB and YidC accumulation levels are regulated. At any rate, optimizing periplasmic protein production yields can also lead to adaptations that make the *E. coli* cell a more efficient protein factory.

It has been shown that the accumulation levels of phage shock protein A (PspA) can increase upon the production of a protein in the periplasm (Champion et al., 2003). PspA is encoded by the phage shock protein (*psp*) operon (*pspABCDE*) which plays a key role in maintaining membrane integrity and the proton-motive force under various stress conditions (Mitchell and Silhavy, 2019). Interestingly, co-production of PspA can enhance both Sec- and Tat-mediated protein export (Champion et al., 2003; DeLisa et al., 2004). It is tempting to speculate that increased PspA accumulation levels can make *E. coli* more fit for efficient periplasmic protein production by maintaining the integrity of the cytoplasmic membrane and the proton-motive force (Mitchell and Silhavy, 2019).

Finally, to facilitate the adaptation of *E. coli* for the production of a protein in the periplasm an approach called global transcriptional machinery engineering (gTME) has been used (McKenna et al., 2019). More specifically, to enhance the production of full length antibodies in the periplasm a mutant library of the principal sigma factor RpoD, a. k.a. σ^70^, was co-produced with the protein. For the sake of clarity, it should be mentioned that the cells still had an intact wild-type *rpoD* gene. Various RpoD variants were isolated that helped to increase full length antibody production in the periplasm. Transcriptome analysis showed that the RpoD variants altered the transcriptomes significantly, but did not really help to reveal why in cells with the RpoD variants production of full length antibodies in the periplasm was enhanced.

## Concluding Remarks and Future Perspectives

Many different strategies have been used to enhance periplasmic protein production yields in *E. coli.* Usually, these are based on changing one parameter or variable at a time. So far, combinatorial screens, *i.e*., screens changing different parameters or variables simultaneously, have hardly been used (Eichmann et al., 2019; Karyolaimos et al., 2019). New technologies and methodologies facilitating large-scale culturing and monitoring protein production levels will greatly facilitate combinatorial screening using different strains, expression vectors, signal peptides, induction regimes, culturing methods and conditions (Krause et al., 2016; Tripathi and Shrivastava, 2019; Kastenhofer et al., 2021). Tunable and independently controlled promoter setups, like the ones of the recently developed “Marionette” strain/expression vector collection, as well as CRISPR-based gene regulators have tremendous potential to facilitate combinatorial co-production screening (Meyer et al., 2019; Reis et al., 2019; Ho et al., 2020). The “Marionette” promoter setups and CRISPR-based gene regulators enable screening for the impact on periplasmic protein production using a myriad of different expression intensities of the gene encoding the recombinant protein and the gene encoding the co-produced component. Usually, one and sometimes two components are co-produced at a time, whereas co-production of multiple (>2) components may actually be needed to further enhance production yields. With the current DNA synthesis and cloning technologies, it should not be difficult to engineer versatile expression vector library setups for multi-component co-production strategies (Hughes and Ellington, 2017). Aforementioned “Marionette” promoter setups and CRISPR-based gene regulators will enable tunable and independently controlled expression of the genes encoding all actors involved. The co-production of components can also have indirect positive effects on periplasmic protein production yields (Ow et al., 2010). Furthermore, it has been shown that (the co-production of) variants of components involved in the protein biogenesis can also help to improve periplasmic protein production yields (Pérez-Pérez et al., 1994; Taw et al., 2021). Isolation of such variants will require cleverly designed screens or selections. Also hosts can be customized for the production of a particular recombinant protein in the periplasm using evolutionary and engineering approaches (Wang et al., 2009; Robertson et al., 2021; Wannier et al., 2021).

It should not be forgotten that our knowledge of gene expression, protein translation, protein targeting, translocation, folding and stability is everything but complete. Furthering our knowledge in these areas may also open up new avenues for enhancing periplasmic protein production yields. Studying *E. coli* cells producing proteins in the periplasm using omics approaches, may help to identify components that can positively and also negatively affect periplasmic protein production yields (*e.g.,* (Ceroni et al., 2015; Reis et al., 2019; Karyolaimos et al., 2020; Mateus et al., 2020; Tan et al., 2020; Rugbjerg et al., 2021)). Such studies may extend the repertoire of components that can be co-produced and used for the isolation of variants as well as genes that can be inactivated to enhance periplasmic protein production yields.

Taken together, different strategies have successfully been used to enhance periplasmic protein production yields in *E. coli* and there appears to be ample room for further improvements. The more information becomes available, the easier it may become to develop predictors and other computer-aided tools that can assist in designing strategies to enhance periplasmic protein production yields (Salis et al., 2009; Orfanoudaki et al., 2017; Almagro Armenteros et al., 2019; Reis and Salis, 2020; Bhandari et al., 2021; Cetnar and Salis, 2021). Finally, we still can learn a lot from how Genentech capitalized on decades of work on the *lac* operon to produce insulin in *E. coli,* but also from its contributions to the field of periplasmic protein production (*e.g.,* (Goeddel et al., 1979; Chang et al., 1987; Joly et al., 1998; Champion et al., 2001; Champion et al., 2003; Chen et al., 2004; Zhou et al., 2016; McKenna et al., 2019)). The many contributions Genentech has made to this field are based on systematically designed studies that show the benefits of standardization and the importance of considering scalability early on.
